# Influence of Different Diets on Growth and Nutritional Composition of Yellow Mealworm

**DOI:** 10.3390/foods11193075

**Published:** 2022-10-04

**Authors:** Anna Bordiean, Michał Krzyżaniak, Marek Aljewicz, Mariusz Jerzy Stolarski

**Affiliations:** 1 Department of Genetics, Plant Breeding and Bioresource Engineering, Faculty of Agriculture and Forestry, University of Warmia and Mazury in Olsztyn, Plac Łódzki 3, 10-724 Olsztyn, Poland; 2 Department of Dairy Science and Quality Management, Faculty of Food Sciences, University of Warmia and Mazury in Olsztyn, ul. Oczapowskiego 7, 10-719 Olsztyn, Poland

**Keywords:** edible insects, small livestock, novel foods, protein, fatty acids, byproducts, bioconversion, *Tenebrio molitor*

## Abstract

Insects are a pathway through which agro-food waste can become a high-quality source of nutrients for both livestock and humans. Yellow mealworm (*Tenebrio molitor* L., Coleoptera: Tenebrionidae) larvae are currently one of the insect species permitted for consumption, and they are reared on a large scale in Europe. This study evaluated the effect of seven diets containing byproducts such as wheat, rye bran, rapeseed meal, rapeseed cake, flax, and milk thistle cakes on the insect’s growth performance, feed conversion ratio (FCR), efficiency of conversion of ingested feed (ECI), nutritional quality of the larvae, and the composition of fats and fatty acids they contained. The lowest FCR based on the fresh and dry basis was 3.32 and 2.01, respectively. The ECI values were statistically different for larvae reared on different experimental diets (mean value 45.7%). As for the nutritional profile, protein and fats ranged from 43.6 to 53.4% d.m. and from 22.3 to 30.0% d.m., respectively. The major fatty acids in all samples were oleic acid (32.97–46.74% of total fatty acids (TFA)), linoleic acid (22.79–38.98% of TFA), and palmitic acid (12.80–17.81% of TFA). This study offers a new opportunity to use and efficiently convert cheap industrial byproducts using yellow mealworms.

## 1. Introduction

Although scientific and technological progress significantly improves people’s lives, it is still predicted that 300 million people will continue to suffer chronic hunger in 2050 due to population growth [1]. Along with the economic growth, there is an increase in prices for agri-food products and higher poverty levels, as well as a decrease in the quality of environmental factors (water, soil, air), exerting strong pressure on climatic conditions. This implies the need to accelerate the implementation of food security measures [2] and to increase the production of food by about 35 to 62% to meet the demand predicted by 2050 [3]. Based on FAO data [4], about one-third of all food produced worldwide becomes waste, which is more than 10% of the world’s total caloric energy consumption. Such waste or byproducts are an important source of nutrients and active compounds, including polyphenols, tannins, flavanols, vitamins, essential minerals, fatty acids, etc. [5]. The bioconversion of these byproducts by insects must be considered from a broader perspective, in the context of a circular economy [6]. Insects not only reduce the volume of biomass, but also use the nutrients it contains to grow and develop, thus becoming an important source of valuable nutrients. They can serve as substitutes for regular feed for livestock [7,8,9,10,11], such as fishmeal and fish oil, and soymeal/soybean, used currently. Insects could also become an alternative source of food, especially for carnivorous species, and contribute to the quality and quantity of nutrients (omega-3 fatty acids), as well as the improved health of fish. There is a growing demand by poultry and livestock farming for other types of animal origin proteins. Currently, nearly 80% of the global agricultural land is used for growing livestock. However, livestock provides less than 20% of the calories used in human diets worldwide [12].

Whereas around 2000 insects at various stages of development are known to be eaten as food all over the world [13], the European Food Safety Authority (EFSA) has issued a list of only 15 edible insect species [14], considered to be novel foods in Europe, according to Regulation 2283/2015 [15]. Based on Regulation 2017/893 [16], proteins from seven insect species were allowed as a feed for non-ruminant farmed animals, other than fur animals. Moreover, in 2017, Switzerland, became the pioneer country in Europe by allowing insects as food [17]. Although insects could be found in some European markets and shops sold as commercial products by specialized companies, they have not been clearly regulated in terms of food safety, nor strictly subjected to existing regulations as a consequence of more permissive transition approaches until they are fully marketable products. However, the first insect-based product that was allowed on the European market in early 2021 was powdered yellow mealworm (*Tenebrio molitor*) [18]; later on, in December 2021, frozen, dried, and powdered yellow mealworm, as well as house cricket (*Acheta domesticus*)— frozen, ground, and dried—and *Locusta migratoria*, were legalized. Yellow mealworm (*Tenebrio molitor* L.) is one of the most reared insect species in Europe [19]. It is known for its ease of handling and good growth, as well as for its nutritional peculiarities. Larvae can process a wide variety of substrates from agricultural and food industries, including wastes from bakery, beer, corn stover, vegetable industries, etc. However, waste from catering (cf. Regulation 1069/2009), former foodstuff that contains fish or meat (cf. Regulation (EC) No 142/2011), or manure and any animal feces (cf. Regulation (EC) 767/2009) are still banned from use in insect rearing [20]. In order to be used as food, the larvae must be grown on organic plant substrates, such as grains and their components (brans, flour, vegetal parts), and a few types of biomasses of animal origin, in compliance with Annex IV of Regulation (EU) No 142/2011. However, the use of plant-based substrates is competitive with human food and animal feed. Therefore, the most preferable in this case could be the cheapest plant byproducts, for example, cereal brans, which are well digested in the guts of many insect species, such as yellow mealworm, considered to be a pest insect in grain production [21].

Fatty acids (FAs) and fats play important biological roles in human health. They contribute to the production of hormones and antibodies, are building blocks of cells and tissues, play a role in the permeability of cells, are used to build nerve tissues, provide energy, and participate in various cellular and molecular processes that take place in the body [22]. Many of these fatty acids are synthesized by the human body (i.e., saturated fatty acids (SFA)), except for some essential polyunsaturated acids (PUFAs), such as linoleic and α-linolenic acids (LA and ALA, respectively), which are precursors of omega-6 and omega-3 fatty acids. The higher omega-3 and omega-6 ratio (or lower n-6 to n-3 ratio), the better is the influence on human health [23]. Fatty acids or their precursors are ingested with food. Mealworms have also been investigated for their fatty acid content. Feed can influence the content and concentration of certain fatty acids, which can later contribute to a balanced human diet.

The main objective of this study was to investigate the growth performance, conversion efficiency, and proximate composition of yellow mealworm larvae grown on diets composed of byproducts from agro-industry, as well the influence of different diets on the fatty acids profile of yellow mealworm larvae.

## 2. Materials and Methods

### 2.1. Experimental Insects

Yellow mealworm larvae (approximately 11th–12th instar) were purchased from a commercial supplier (CRICKETSFARM) specializing in growing insects in Poland. The rearing methodology of mealworm larvae is based on our previous research described in Bordiean et al. 2020 [24]. The insects were maintained under laboratory conditions (relative humidity: 55–60%, air temperature: 28 °C, photoperiod: 12L:12D). The photoperiod time was designed based on other studies [25,26]. The purchased mealworm larvae were reared to obtain a colony of mature individuals. The mealworms were fed ad libitum with chicken feed, which was composed of corn, wheat gluten feed, wheat, soybean meal (genetically modified), calcium carbonate, vegetable oil, sodium chloride, and rapeseed meal. Chicken feed (consisting of 18.05% crude protein, 3.66% crude fat, 3.32% fiber, 13.96% ash, 4.10% calcium) had been previously milled to the size of 3 mm. The larvae were also provided with fresh carrots three times a week. The insects were kept in plastic containers (35 × 23 × 13 cm) with aeration holes on the sides, and closed with a lid. The temperature and relative humidity were monitored to verify the parameters in the boxes. After a few weeks, the first pupa appeared, and a new adult generation was obtained after another 6–7 days. The adults were placed in containers fitted with mosquito mesh on the bottom that allowed the eggs (with small feed particles) to fall into an egg collection container. The eggs were collected in a short period of time (12 days) to prevent large age differences between larvae. The hatching of collected eggs was estimated to last 7–10 days after the end of the egg collection period. Newly hatched larvae were fed ad libitum with chicken feed. This procedure allowed the larvae to grow to a size enabling their easy and safe collection for the main experiment. The experiment started when larvae were approx. 35 days (five weeks) old.

### 2.2. Diet Preparation, Larval Growth, and Measurements during the Experiment

Prior to the experiment, some agro-industry byproducts were selected for testing. The experimental dietary mixes were composed of wheat bran (1), used as the control feed (WB 100), (2) rye bran (RB), (3) rapeseed meal (from hot pressing and chemical extraction by solvents) (RM), (4) rapeseed cake (RC), (5) flax cake (FC), and (6) milk thistle (*Silybum marianum* L. (Gaertner); Asteraceae) cake (SMc). All cakes were cold-pressed at temperatures of 40–45 °C in a small oil-production facility.

The diets were prepared by grinding the ingredients through 6 mm (RC and FC pellets) and 3 mm (SMc pellets) sieve openings. All diets were then sifted through a 300 μm sieve to remove the smallest particles of a size similar to the size of larvae faeces. Subsequently, the proximate analysis of all the feeds was determined, including dry matter, ash, crude protein, crude fat, crude fiber, and nitrogen free extract (NFE) (Table 1). The NFE estimates non-fibrous carbohydrates, such as soluble mono- and oligosaccharides, and starches. The NFE (and other components) was calculated according to Equation (1):

NFE (% dry mass) = 100% − [CP (% d.m.) + CF (% d.m.) + CFb (% d.m.) + Ash (% d.m.)],(1)

where CP is crude protein, CF is crude fat, and CFb is crude fiber contents in the diets. Experimental feeds were mixed with wheat bran in the proportion of 70% of WB and 30% of meal or cake, except for rye bran, which was used as a separate diet (100%). In this way, seven types of diets were obtained. The proximate composition of the experimental diets and control were estimated as percentage ratios (by weight) (Table 2). The diets were stored at −20 °C until the start of the experiment.

### 2.3. Larvae Rearing Conditions

The experiment began when mealworm larvae were 35 days old. They foraged ad libitum and undisturbed on chicken feed (oviposition substrate). Around 6.7 g of larvae (trapped by using carrots and sieves) were collected for each experimental diet. The average weight of one larva was estimated to be 0.5 mg, as determined from weighting 20 live larvae (in three replicates). This stage was transferred to separate boxes (35 × 23 × 13 cm). Based on the mealworm individual larval weight, it was estimated that there were approximately 1343 larvae per diet/box. Insects were fed the experimental diets (from 20 g to 150 g) and carrots (from 10 g to 40 g). Wheat bran was chosen as a control diet, based on the results of our previous study, in which this feed resulted in the highest weight of larvae and the shortest larval development period [24]. Feed was supplied weekly. To maintain the constant humidity in the boxes, carrots were added twice per week. The supplied amounts of feed and carrots were recorded as well, being adjusted weekly according to mealworm growth. Weekly frass was removed by sieving with 300 μm openings, and the larval development parameters were monitored by weighing 10 mealworms completely randomly, and then returning them to the container. Meanwhile, dead insects were removed to prevent cannibalism or altering the feed quality. When the first pupae were observed, the mealworms were separated with a 2 mm sieve and left to purge for 24 h. Afterwards, all larvae were weighed and sacrificed by freezing at −20 °C. Then, the larvae were dried at 60 °C until the constant weight was achieved, approximately 72 h.

### 2.4. Feed Conversion Ratio

Determination of the average of both the live larvae weight gain (LWG) (based on difference in weight from the previous week’s measurements), as well as individual weight (IW) (mg fresh matter (f.m.)), was conducted weekly.

Feed conversion efficiency was calculated at the end of the experiment as the feed conversion ratio (FCR) and efficiency of conversion (ECI) of ingested feed. The ECI (Equation (2)) was calculated based on the method of Waldbauer [27]:

ECI = (final weight/weight of ingested feed) × 100 (%),(2)

and expressed on a dry matter basis. The feed conversion ratio (FCR) was calculated (Equation (3)) by dividing the weight of ingested feed by the total mean individual weight gain [28], and expressed on the fresh (FCR_ff_) and dry matter basis (FCR_df_) of the feed and carrots:

FCR = weight of ingested food/weight gained(3)

### 2.5. Nutritional Analysis

The proximate analysis of feed and larvae included crude protein (CP), crude fat (CF), crude fiber (CFb), moisture, and ash content. The crude protein content was measured using the Kjeldahl method; crude fat and fat extraction (for larva only) were determined using Soxhlet extraction, with petroleum ether as a solvent. Fiber was analysed according to the PN-EN ISO 13906:2009 by using ANKOM A200 (Macedon, NY, USA), and ash content was assessed by using an automatic ELTRA TGA-THERMOSTEP analyser (Neuss, Germany), according to the PN-EN ISO 16948:2015-07 and PN-EN ISO 16994:2016-10, respectively. The nitrogen to protein conversion factor for insects was 5.41, based on Boulos et al. [29].

Fatty acid methyl esters (FAME) were prepared according to Zaderimowski and Sosulski [30], with some modification. Approximately 10 μg of the oil sample was placed into a screw-capped glass tube; then, 2 mL of the methylating mixture (methanol: chloroform: sulfuric acid—100:100:1, *v*/*v*/*v*) was added. Subsequently, the tube was purged with nitrogen for 15 s before sealing. Methylation was carried out by heating the tubes at 70 °C for 2 h. The methylating texture was evaporated with a Centrivap rotary vacuum evaporator (Labconco, Kansas City, MO, USA); then the methyl esters were dissolved in 2 mL of n-hexane (Sigma-Aldrich, St. Louis, MO, USA) and vortexed for 15 s.

The methyl esters were analysed by gas chromatography with mass spectrometry using a GC-MS QP2010 PLUS system (Shimadzu, Kyoto, Japan), according to the parameters described by Czaplicki et al. [31]. Separation was performed on a BPX70 (25 m × 0.22 mm × 0.25 μm) capillary column (SGE Analytical Science, Victoria, Australia), with the following analytical conditions and programing: helium as the carrier gas at a flow rate of 0.9 mL per min; the temperature of the ion source was 240 °C, with heating from 150 °C to 180 °C at the rate of 10 °C for 1 at the rate of 30 °C for 1 min, and then 10 min of holding; the electron energy was 70 eV. The total ion current (TIC) mode was used in the 50–500 *m*/*z* range, and compounds were identified based on their mass spectra compared with mass spectral libraries (NIST08 library, Shimadzu, Kyoto, Japan).

### 2.6. Statistical Analysis

All statistical analyses were performed with the Statistica 13 software package (TIBCO Software Inc., Palo Alto, CA, USA). Collected data were subjected to one-way analysis of variance (ANOVA). Significant differences between means were determined by the Tukey’s (HSD) multiple tests at α = 0.05.

## 3. Results and Discussion

### 3.1. Growth Performance and Feed Conversion Ratio

The result of one-way ANOVA showed no significant difference in the final weight and FCR_ff_ of mealworm reared on different diets (Table 3).

**Figure 1:**
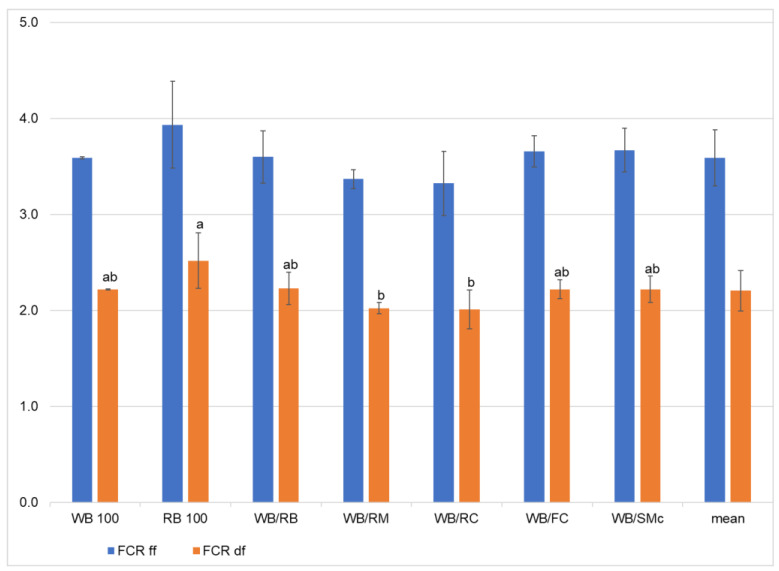
Feed-conversion ratio (FCR) on the fresh (ff) and dry matter basis (df), depending on the diet; WB (wheat bran), RB (rye bran), WB/RB (wheat bran/rye bran), WB/RM (wheat bran/rapeseed meal), WB/RC (wheat bran/rapeseed cake), WB/FC (wheat bran/flax cake), WB/SMc (wheat bran and *Silybum marianum* cake); the letters a, b, etc., show statistically homogenous groups (Tukey’s test at *p* < 0.05); bars represent standard deviation.

FCR_df_ based on the dry matter of feed and carrots was significantly differentiated by the experimental diets (Table 3, Figure 1). The lowest values for this feature were measured for larvae fed on mixtures with rapeseed (WB/RC and WB/RM; 2.01 and 2.02, respectively). The highest FCR_df_ (2.52) was found for mealworms fed on the RB 100 diet, while the other four diets formed an intermediate group between the best and worst FCR with values from 2.22 (WB 100, WB/FC, WB/SMc) to 2.23 for WB/RB. A slightly lower FCR (from 1.57 to 2.08) was obtained in another study for mealworm larvae feed on different mixes that contained chicken feed and some industrial byproducts (rapeseed meal, wheat bran, and willowleaf sunflower, pure or mixed with chicken feed in different proportions—25, 50, and 75%) [24].

While FCR_df_ was significantly differentiated by the experimental diets, the FCR_ff_ based on the fresh matter was not, amounting to 3.59 on average (Table 3, Figure 1). The expression of the FCR based on both the dry and fresh matter is important to correctly estimate the nutritional needs to obtain mealworms. Often, however, they must go through a processing stage to reduce their microbial load, extend the storage period, and preserve their nutritional features (prevent fat oxidation). The FCR expressed on the fresh weight basis in the present study corresponds to the values from another study [25]. The FCR values depend mostly on the nutritional quality and moisture content (especially of carrots) of the byproducts used, being extremely high, from 4.35 [32] up to 19.1 for low protein content high-fat diets [26]. The consumption of carrots is very often excluded from the FCR and ECI calculations [24,25,26] because of their high-water content and low concentration of nutrients. However, the present study took into account the dietary carrot, even though the FCR values would be increased. It is important to mention that the mealworms were supplied with carrots twice per week, so they did not have a surplus of carrots. In fact, carrot was added to maintain the recommended humidity in the containers and to improve the conversion of nutrients from feed.

**Figure 2:**
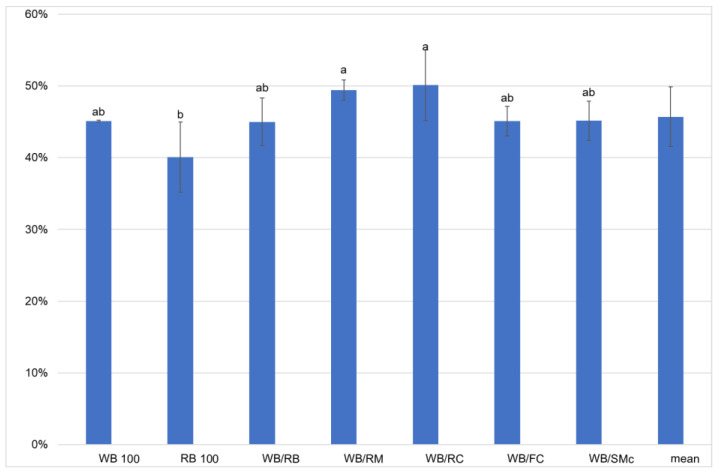
Efficiency of conversion of ingested feed (ECI), depending on the experimental feed; WB (wheat bran), RB (rye bran), WB/RB (wheat bran/rye bran), WB/RM (wheat bran/rapeseed meal), WB/RC (wheat bran/rapeseed cake), WB/FC (wheat bran/flax cake), WB/SMc (wheat bran/*Silybum marianum* cake); the letters a, b, show homogenous groups (Tukey’s test at *p* < 0.05); bars represent standard deviation.

The ECI (efficiency of ingested feed) values were statistically different for mealworm larvae fed on the experimental diets. The lowest ECI value (40.1%) was found for mealworms fed on the RB 100 diet, which was included in homogeneous group b. The higher ECI values (49.4 and 50.1, group a) were found to be for larvae fed on diets that included rapeseed meal and rapeseed cake, respectively (Table 3, Figure 2). The higher the ECI, the better the conversion of ingested feed by insects. The protein content of feed seems to play an important role in ensuring a higher ECI. Thus, the lowest protein content (15.04%) d.m. of the RB 100 diet resulted in the lowest ECI, while the high protein content diets (22.8 and 20.4 % d.m. of RM and RC diets, respectively) contributed to the highest ECI of the mealworms. The same influence of the protein content of diets on ECI has been reported by other researchers [24,25,26]. Moreover, it is obvious that there is a negative correlation between FCR and ECI, and usually a lower FCR corresponds to a higher ECI [25], so that the ingested feed is converted more efficiently by insects. The ECI values determined in the present study are higher than those reported by Van Broekhoven et al. [25], where the ECI ranged from 16.76 to 28.93% for mealworm larvae fed on an LPHS (low protein and high starch) and HPHS (high protein and high starch) diet, respectively. Lower values are reported by Oonincx et al. [26], where the ECI ranged from 7 to 21% for insects being fed on different byproduct diets (LPLF—low protein and low fat, and a commercial control diet, respectively). Additionally, a very low ECI was achieved for mealworms fed on willowleaf sunflower biomass (5.9%) [24]. Similar ECI values to those obtained in our study were reported by Zhang et al. [33]. Thus, the ECI of yellow mealworms were 36, 42, 46, and 56% when the larvae were grown on spirit distillers’ grains, highly denatured soybean meal, mushroom spent corn stover, and wheat bran, respectively.

**Figure 3:**
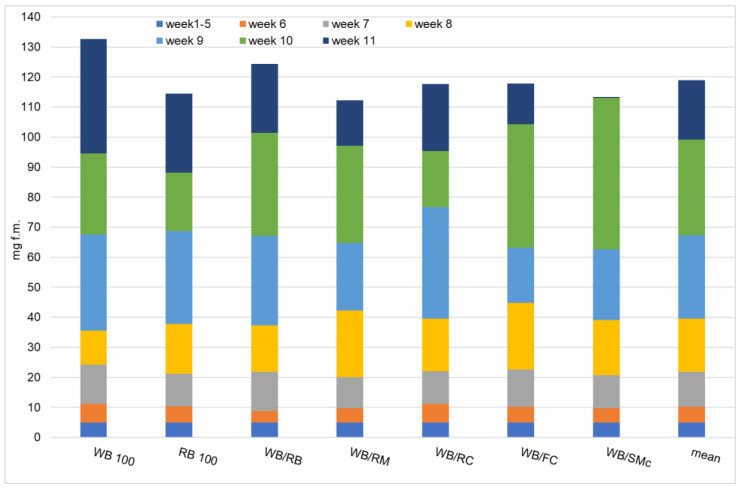
Individual larval weight gain and the final weight (on wet weight basis) of yellow mealworm larvae depending on the experimental diet; WB (wheat bran), RB (rye bran), WB/RB (wheat bran/rye bran), WB/RM (wheat bran/rapeseed meal), WB/RC (wheat bran/rapeseed cake), WB/FC (wheat bran/flax cake), WB/SMc (wheat bran/*Silybum marianum* cake).

The weight gain of individual mealworm larvae (LWG) (Figure 3) was measured weekly in order to observe their growth on different diets. The estimated LWG during the first five weeks was 4.9 mg. After the larvae were fed on experimental diets, their LWG differed slightly between the diets. Thus, after the sixth week, their LWG ranged between 3.9 and 6.31 mg for larvae fed on WB/RB and WB/RC, respectively. In the following week, their LWG ranged between 10.33 and 13.13 mg for larvae grown on WB/RM and WB 100, respectively. After eight weeks, the LWG changed between the diets again; some larvae gained more weight and others less, with the LWG ranging from 11.30 to 22.10 mg for mealworms fed on WB 100 and WB/RM, respectively. At week 10, the lowest gain (18.63 mg) was achieved by the larvae fed on WB/RC. The most spectacular growth based on LWG was recorded for larvae fed on WB/SMc, where the LWG reached 50.40 mg in the 10th week. However, for the consecutive weeks’ measurements, the larvae fed on the WB/SMc showed the lowest LWG (0.30 mg), meaning that the insects had completed their development and were ready for metamorphosis. The final larval weight for all the tested diets was statistically the same and amounted to 118.93 mg f.m. on average. However, the highest final weight of a single larva was 132.6 mg f.m. for insects reared on the control diet (WB 100). A slightly lower value was found for larvae reared on the WB/RB diet (124.4 mg f.m.). The lowest fresh weight was found for larvae fed with WB/RM (112.3 mg f.m.). Although all diets contained wheat bran (except RB 100), even a 30% share of another byproduct could significantly influence the growth and development of the insects. Because insects were fed on different diets, they may also have had a different rhythm of the growing rate. Therefore, feeding on a diet not sufficiently adapted to their needs means earlier pupation and a lower weight gain. From this point of view, the mealworms no longer gain weight in the last stages of development [34,35,36], preparing their bodies and reserves for the next development stage.

### 3.2. Proximate Analysis

The proximate composition of an 11-week-old yellow mealworm depended on the type of a tested diet (Table 4). The proximate analysis was carried out based on dry weight. Significantly, the highest dry matter content (33.3%) was found in larvae fed on RB 100 (homogeneous group a), and the lowest (30.3%) in larvae grown on WB/SMc (homogeneous group d). The dry matter content of another species of mealworm (lesser mealworm *Alphitobius diaperinus*) fed on chicken feed alone or enriched with flax seed oil was similar (33–36%) [37]. The ash content did not differ significantly and ranged from 3.64 to 5.19% d.m. of larvae fed on RB 100 and WB/FC, respectively. The fiber content was 7.08% d.m. on average, and differences between the diets were also insignificant. Chitin is the main component of dietary fiber, and its amount in mealworm larvae is very small and difficult to estimate due to differences between various analytical methods [38]. In a recent study, it has been estimated that mealworm larvae contain 4.92% of chitin in their entire body [39]. Additionally, processing methods, such as different drying approaches, can influence the proximate composition of mealworms [40,41]. Thus, the ash and crude fiber content of the mealworms were comparable in quantity to results obtained in other research on processed or unprocessed mealworms [40] and on mealworms fed on substrates that contained 90% different organic wastes (vegetable and garden waste, cattle and horse manure) and 10% chicken feed [42].

The most important nutritional components of insects are proteins and fats (Table 4). In the present study, the nitrogen-to-protein conversion factor (5.41) proposed by Bolous et al. [29] is lower than that used (6.25) in most of the studies regarding insect protein content, being slightly overestimated for the protein content of insects [38]. Thus, it is possible that the protein content of mealworm larvae is lower than that measured in some other studies. The significantly highest protein content (53.4% d.m.) was assayed in the insects fed on WB/FC. A slightly, but statistically significantly lower, value (52.5% d.m.) was found in larvae grown on WB/RM. The lowest protein content of the WB 100, RB 100, and WB/RB diets resulted in a lower protein content in the insects fed on these diets. The insects raised on wheat bran had 47.9% d.m. of protein and were classified in homogeneous group f. Generally, the protein content of larvae nearly doubled compared to the protein values of the diets. Such results have also been reported for mealworm larvae and crickets in other studies [26,43]. It is important to mention that the quality rather than the amount of protein from feed plays a significant role in mealworm protein content [43]. Although it appears that the higher protein content of feed may increase the protein content of insects, some deviations from this rule have also been observed. This occurred in our experiment. For instance, when the protein content in some diets was nearly the same (WB/RC—20.4% and WB/FC—20.2%) (Table 2, see Material and Methods section), the protein content of the larvae consuming these diets differed significantly (50.9 and 53.4%, respectively, Table 4), so the larvae were included in different homogeneous groups (d and a, respectively). Thus, a high protein content in the feed does not necessarily ensure a high protein content of the larvae. This suggests that even poor-quality feed [44] in terms of protein (WB/SMc—17.5% d.m. crude protein, Table 2) can be used to grow insects, and it still offers an opportunity to obtain insects with a significant concentration of protein (52.0% d.m.). The same scenario was found in another study, where the MSCS diet (mushroom spent corn stover) had 3.90% of CP; HDSM (highly denatured soybean meal) had 43.18% CP; SDG (spirit distillers’ grains) and wheat bran both had 16.98% CP. After a 60-day rearing, mealworms contained 75.25, 74.43, 70.10, and 69.93% d.m. CP, respectively [33].

The crude fat content of yellow mealworm larvae differed significantly between the diets used (Table 4). The highest fat content (30.0% d.m.) was found in larvae raised on RB 100. The lowest value of crude fat was 22.3% d.m. in insects fed on WB/RM. It was observed that higher fat content diets (WB/RC—7.86% d.m. and WB/FC—7.03% d.m., Table 2) did not influence the fat content of the larvae (28.6 and 24.4% d.m., respectively), and larvae fed on these diets fell into statistically different homogeneous groups (b and d). The fat content in insects is rather influenced by NFE amount in the tested diets (Table 2). In the study in [45], it was found that around 90% of the body fat of insects is triglycerides, which are synthesized from dietary carbohydrates, fatty acids, or proteins. The same authors mentioned that the conversion of carbohydrates from diets to lipids in body fat was documented as well. The crude fat contents of larvae fed on experimental diets in present study are comparable with data reported from other studies [25,26,40]. Higher values of the total fat content (39.75–48.31% d.m.) of mealworms were obtained in a study of mealworms fed on different flours and byproducts (fed on ventilated oats ground to flour, corn and wheat flours, chickpea flour, bread, and beer yeast) [46]. Combining different processing methods of mealworm drying (e.g., blanching, convective drying, freeze drying) of larvae can alter the fat content of mealworms fed on chicken feed [41]. Insects bred in captivity for commercial purposes have a higher fat content than those bred in the wild, as they lose some of their energy resources in search of feed [47,48]. Fat accumulation in the mealworm body occurs mostly in the prepupal stages, when the larva body is preparing for the next metamorphosis stage [36,40].

### 3.3. Fatty Acid Profile

Yellow mealworm fats are an important contributor to their nutritional value. The fatty acid profiles of larvae fed on different diets are shown in Table 5 and are in accordance with the profiles found in other studies. Depending on the diet on which the insects were raised, there are some differences in the qualitative and quantitative composition of the TFA. The main components of mealworm fatty acids are oleic, linoleic, and palmitic acids. The highest fraction of SFA is composed of palmitic acid, as its share ranged from 12.80 to 17.81% TFA for larvae grown on WB/RC and RB 100 diets, respectively. The same levels are reported in the literature [25,26,33,49].

Insect fats are valuable in terms of unsaturated fatty acids (UFA). In all samples, the highest fraction of fats belonged to oleic acid (C18:1 n-9) and ranged from 32.97 to 46.74% TFA for larvae raised on WB/FC and RB 100, respectively. These values are higher than the ones reported for mealworms (20.6–35.6%) fed on highly denatured soybean meal, spirit distillers’ grains, and mushroom spent corn stover mixed with wheat bran [33]. Another fatty acid fraction that differed quantitatively is linoleic acid (C18:2 n-6), which amounted to 22.79–38.98% TFA for the RB 100 and WB/SMc diets, respectively. Lower values were reported for insects fed on diets composed of different byproducts (maize distillers’ dried grains with soluble, spent grains, beer yeasts, bread remains, cookie remains, steam potato peelings) [25]. An important share (17% TFA) of α-linolenic acid (C18:3) has been found in the fat of larvae fed with WB/FC compared to the values found in other samples, where it ranged from 1.11 to 2.60% TFA for RB 100 and WB/RC, respectively. The enrichment of diets with flax flour increased also the α-linolenic acid share in mealworm fat in another research [46,49].

The PUFA/SFA and ω-6/ω-3 ratios are important to achieve a healthy diet. The most recommended ω-6/ω-3 ratio must be close to 4:1–5:1 and should not exceed 10:1 [50]. The total content of unsaturated fatty acids in larvae ranged between 73.65 and 82.19% of TFA in RB 100 and WB/FC, respectively. The diets that contained flax cake (WB/FC) significantly increased the percentage of α-linolenic acid up to 17% TFA, compared to the values determined in insects raised on the other diets, which varied only slightly, from 1.11 to 2.60% TFA for RB 100 and WB/RC, respectively. Moreover, the diet with flax cake decreased the value of stearic acid (C18:0) in mealworms (0.24% TFA), compared to the remaining diets. The high share of α-linolenic acid that is an ω-3, considerably reduced the ω-6/ω-3 ratio to 1.71 and increased the PUFA/SFA ratio to 2.79 in larvae bred on WB/FC. The insects diets enriched with flaxseed oil (up to 4%) or flax flour (10%) [51] significantly reduced the ω-6/ω-3 ratio, a result observed in mealworm larvae, as well as other insects [37,49]. The ω-6/ω-3 ratio reported elsewhere (15.90–21.03) [33] is comparable with the ratio (11.44 to 25.99) found in the present study for all the diets, excluding WB/FC. Our research found that the MUFA and PUFA content in the mealworm larvae fat was influenced by the composition of the diets. The highest share of MUFA was found in mealworms fed with the RB 100 diet (49.35% TFA), and the highest share of PUFA was found in fat from larvae fed on WB/FC (46.63% TFA) and WB/SMc (41.15% TFA). Flax and *S. marianum* oil are known for their high content of PUFA, which resulted in the highest content of these fatty acids in the mealworm larvae [37,49,52,53].

Although the main component of the mixed diets is wheat bran, the remaining ingredients (30% per diet) play a major role in the fatty acid composition in mealworm larvae. Therefore, diets with a low amount of ω-3 can reduce this amount in insects as well. Even the inclusion of 1% flaxseed oil or 10% flax (linseed) flour (rich in ω-3) in the diets of mealworms can considerably improve the ω-6/ω-3 ratio [37,49]. It was also found that carrot in the diets could decrease this ratio [26].

## 4. Conclusions

Our study showed that commonly available byproducts from the agro-industrial sector could successfully be used as feed for rearing high-quality yellow mealworm larvae. We found that the tested diets influenced the protein and fat content of the larvae. The protein content of insects was not necessarily related to the quantity of the protein in a diet, but the quality of feed proteins (amino acids) could play a significant role in the mealworm protein content and quality. Therefore, this issue requires further investigation. We also found that the lower protein content in the diet, the higher the fat content in the larvae. Our results confirmed that yellow mealworms contained a large amount of unsaturated fatty acids, especially oleic acid, linoleic acid, and a significant amount of saturated palmitic acid. We also found that the addition of flax cake resulted in the most balanced proportion of fatty acids (ω6/ω3 ratio). Therefore, diets could be enriched with flax oil in order to obtain insect products with the best ω6/ω3 ratio. Based on the tested features, such as FCR and ECI, one can suggest that the best diets for mealworm growth were those that contained rapeseed meal (WB/RM) and rapeseed cake (WB/RC). Furthermore, this study supplements the list of feeds and feed ingredients potentially used in mealworm farming. These byproducts can be used by insect producers, due to their local availability and affordable prices. In addition, they fit into the current policy of the European Union (Green Deal) by promoting short supply chains and the circular economy. In order to facilitate the bioconversion of nutrients from various byproducts by mealworms, it is necessary to adapt different combinations of byproducts as feed materials in an insects’ diet. This could result in optimal insect growth, a low feed conversion ratio, the high efficiency of the conversion of ingested feed, and the optimal composition of ingredients obtained for animal or human nutrition.

Moreover, in this study, we found that methodology could be improved, which will allow researchers to obtain more reliable results. For example, increasing the number of larvae per replicate could result in more accurate data regarding the weight gain or survival, and more accurate conclusions could be drawn regarding the influence of a specific feed on the yellow mealworm larvae. Furthermore, more research should be done for the selection of an optimal amount and form of water supplied to the insects (e.g., as vegetables, fruits, or hydrogel) and on the influence of the tested diets on the other insect stages (pupa and adult), their weight, survival, oviposition rate, and survival of the next larvae generation in subsequent studies.

## Figures and Tables

**Table 1:** Main components of agro-industrial byproducts used for experimental diets.

No.	Substrate	Moisture (%)	Ash (% d.m.)	Crude Protein (% d.m.)	Fiber Content (% d.m.)	Crude Fat (% d.m.)	NFE (% d.m.)
1.	Wheat bran (WB)	12.46	5.63	17.34	8.29	4.12	64.62
2.	Rye bran (RB)	11.41	4.16	15.04	3.79	2.76	74.25
3.	Rapeseed meal (RM)	11.88	7.33	35.41	10.57	1.88	44.81
4.	Rapeseed cake (RC)	9.88	5.83	27.68	16.74	16.58	33.17
5.	Flax cake (FC)	8.73	5.47	27.01	5.47	13.82	48.23
6.	Milk thistle cake (SMc)	9.61	5.41	17.98	26.23	3.62	47.77

**Table 2:** Proximate composition of experimental diets and control.

No.	Diets	Moisture (%)	Ash (% d.m.)	Protein (% d.m.)	Fiber (% d.m.)	Crude Fat (% d.m.)	NFE (% d.m.)
1	WB 100 (control)	12.46	5.63	17.34	8.29	4.12	64.62
2	RB 100	11.41	4.16	15.04	3.79	2.76	74.25
3	WB/RB	12.1	5.2	16.6	6.9	3.71	67.51
4	WB/RM	12.3	6.1	22.8	9.0	3.45	58.68
5	WB/RC	11.7	5.7	20.4	10.8	7.86	55.18
6	WB/FC	11.3	5.6	20.2	7.4	7.03	59.70
7	WB/SMc	11.6	5.6	17.5	13.7	3.97	59.27

Diet abbreviations: WB (wheat bran), RB (rye bran), WB/RB (wheat bran/rye bran), WB/RM (wheat bran/rapeseed meal), WB/RC (wheat bran/rapeseed cake), WB/FC (wheat bran/flax cake), WB/SMc (wheat bran/*Silybum marianum* cake).

**Table 3:** Results of one-way ANOVA.

Source of Variation	FCR_df_	FCR_ff_	ECI	Final Weight	Dry Matter	Ash	Crude Fiber	Crude Protein	Crude Fat
df	6								
F	3.22	1.82	3.18	2.20	20.1	2.87	0.658	2463	130
p	0.03	0.17	0.035	0.10	<0.001	0.05	0.68	<0.001	<0.001

Legend: FCR_df_—Feed Conversion Ratio based on dry feed weight, FCR_ff_—Feed Conversion Ratio based on fresh feed weight, ECI—efficiency of conversion of ingested feed; significant parameters are shown in bold.

**Table 4:** Proximal composition (% on dry weight basis) of 11-week-old mealworm larvae grown on experimental diets.

Feed	Dry Matter (%)	Ash (%)	Fiber Content (%)	Crude Protein (%)	Crude Fat (%)
WB 100	31.7 ± 0.31 ^bc^	3.80 ± 0.08	6.85 ± 0.32	47.9 ± 0.10 ^f^	26.1 ± 0.82 ^c^
RB 100	33.3 ± 0.15 ^a^	3.64 ± 0.05	7.12 ± 0.63	43.6 ± 0.02 ^g^	30.0 ± 0.47 ^a^
WB/RB	31.6 ± 0.15 ^bc^	4.00 ± 0.09	7.12 ± 0.42	49.3 ± 0.02 ^e^	27.5 ± 0.69 ^bc^
WB/RM	32.1 ± 0.25 ^b^	3.85 ± 0.06	7.06 ± 0.34	52.5 ± 0.06 ^b^	22.3 ± 0.09 ^e^
WB/RC	32.3 ± 0.77 ^ab^	3.81 ± 0.07	6.79 ± 0.59	50.9 ± 0.19 ^d^	28.6 ± 0.52 ^b^
WB/FC	30.6 ± 0.30 ^c^	5.19 ± 1.18	7.31 ± 0.31	53.4 ± 0.11 ^a^	24.4 ± 0.40 ^d^
WB/SMc	30.3 ± 0.46 ^d^	4.83 ± 1.11	7.29 ± 0.25	52.0 ± 0.19 ^c^	24.5 ± 0.23 ^d^

Diet abbreviations: WB (wheat bran), RB (rye bran), WB/RB (wheat bran/rye bran), WB/RM (wheat bran/rapeseed meal), WB/RC (wheat bran/rapeseed cake), WB/FC (wheat bran/flax cake), WB/SMc (wheat bran/*Silybum marianum* cake); mean of replicates (*n* = 3) ±standard deviation; superscript letters a–g, show homogenous groups (Tukey’s test at *p* < 0.05) between insects fed on different experimental diets.

**Table 5:** Fatty acid profile in the total lipids (% of total identified fatty acid TFA) of mealworm larvae fed on different diets.

Common Name		WB 100 *	RB 100 *	WB/RB	WB/RM *	WB/RC *	WB/ FC *	WB/SMc
Lauric acid	C12:0	0.22	0.26	0.23	0.19	0.14	0.14	0.18
Tridecanoic acid	C13:0	0.05	0.04	0.05	0.05	0.04	0.04	0.05
Myristic acid	C14:0	2.55	3.29	2.67	2.19	1.71	1.91	2.27
Myristoleic acid	C14:1	0.12	0.15	0.13	0.12	0.13	0.07	0.10
	C14:2	0.12	0.13	0.13	0.14	0.10	0.08	0.11
Pentadecanoic acid	C15:0	0.15	0.11	0.14	0.21	0.13	0.13	0.16
Pentadecanoic acid	C15:1	0.07	0.06	0.06	0.14	0.08	0.08	0.08
Palmitic acid	C16:0	17.33	17.81	17.25	16.26	12.80	14.25	15.44
Palmitoleic acid	C16:1 n (7 + 9)	2.12	1.91	2.42	2.27	2.08	1.30	1.33
Hexadecadienoic acid	C16:2 n-6,9	0.48	0.27	0.40	0.55	0.50	0.39	0.51
Margaroleic acid	C17:1	0.14	0.13	0.13	0.74	n.d.	0.52	0.63
Stearic acid	C18:0	2.43	2.33	2.32	2.50	2.21	0.24	3.17
Oleic	C18:1 n-9	36.53	46.74	39.58	39.32	46.34	32.97	33.46
Vaccenic	C18:1	0.23	0.38	0.29	0.34	0.31	0.54	0.26
Linoleic acid	C18:2 n-6	35.55	22.79	31.66	31.73	29.75	29.12	38.98
α-linolenic acid	C18:3	1.73	1.11	2.33	1.86	2.60	17.00	1.50
Arachidic acid	C20:0	0.08	0.14	0.11	0.07	0.08	n.d.	0.16
Gondoic acid	C20:1 n-9	0.09	n.d.	0.10	0.11	0.16	0.09	0.12
Eicosadienoic acid	C20:2 n-6	0.06	n.d.	n.d.	n.d.	n.d.	0.04	0.05
SFA (% TFA)		22.79	23.96	22.77	21.45	17.11	16.71	21.42
MUFA (% TFA)		39.29	49.35	42.71	43.03	49.08	35.56	35.97
PUFA (% TFA)		37.93	24.30	34.52	34.27	32.95	46.63	41.15
UFA (% TFA)		77.21	73.65	77.23	77.30	82.03	82.19	77.12
SFA/UFA		0.30	0.33	0.29	0.28	0.21	0.20	0.28
PUFA/SFA		1.66	1.01	1.52	1.60	1.93	2.79	1.92
ω6/ω3 (n6/n3)		20.55	20.62	13.56	17.11	11.44	1.71	25.99

SFA (saturated fatty acids), MUFA (monounsaturated fatty acids), PUFA (polyunsaturated fatty acids), UFA (unsaturated fatty acids) = MUFA + PUFA; n.d.—not detected. Diet abbreviations: WB (wheat bran), RB (rye bran), WB/RB (wheat bran/rye bran), WB/RM (wheat bran/rapeseed meal), WB/RC (wheat bran/rapeseed cake), WB/FC (wheat bran/flax cake), WB/SMc (wheat bran/*Silybum marianum* cake); * mean of two replicates (*n* = 2).

## Data Availability

The datasets used and/or analyzed during the current study are available from the corresponding author on reasonable request.
